# A novel approach to neuraxial anesthesia: application of an automated ultrasound spinal landmark identification

**DOI:** 10.1186/s12871-019-0726-6

**Published:** 2019-04-16

**Authors:** Ting Ting Oh, Mohammad Ikhsan, Kok Kiong Tan, Sultana Rehena, Nian-Lin Reena Han, Alex Tiong Heng Sia, Ban Leong Sng

**Affiliations:** 1 Department of Women’s Anaesthesia, KK Women’s and Children’s Hospital, Singapore, Singapore; 2 Department of Electrical and Comupter Engineering, Faculty of Engineering, National University of Singapore, Singapore, Singapore; 3 Center for Quantitative Medicine, Duke-NUS Medical School, 8 College Road, Singapore, Singapore; 4 Division of Clinical Support Services, KK Women’s amd Children’s Hospital, Singapore, Singapore; 5 Anesthesiology and Peroperative Sciences Academic Clinical Program, Duke-NUS Medical School, 8 College Road, Singapore, Singapore

**Keywords:** Automated, Ultrasound, Spinal Anaesthetics: anatomy, Neuraxial anesthesia

## Abstract

**Background:**

Neuraxial procedures are commonly performed for therapeutic and diagnostic indications. Currently, they are typically performed via palpation-guided surface landmark. We devised a novel intelligent image processing system that identifies spinal landmarks using ultrasound images. Our primary aim was to evaluate the first attempt success rate of spinal anesthesia using landmarks obtained from the automated spinal landmark identification technique.

**Methods:**

In this prospective cohort study, we recruited 100 patients who required spinal anesthesia for surgical procedures. The video from ultrasound scan image of the L3/4 interspinous space in the longitudinal view and the posterior complex in the transverse view were recorded. The demographic and clinical characteristics were collected and analyzed based on the success rates of the spinal insertion.

**Results:**

Success rate (95%CI) for dural puncture at first attempt was 92.0% (85.0–95.9%). Median time to detection of posterior complex was 45.0 [IQR: 21.9, 77.3] secs. There is good correlation observed between the program-recorded depth and the clinician-measured depth to the posterior complex (r = 0.94).

**Conclusions:**

The high success rate and short time taken to obtain the surface landmark with this novel automated ultrasound guided technique could be useful to clinicians to utilise ultrasound guided neuraxial techniques with confidence to identify the anatomical landmarks on the ultrasound scans. Future research would be to define the use in more complex patients during the administration of neuraxial blocks.

**Trial registration:**

This study was retrospectively registered on clinicaltrials.gov registry (NCT03535155) on 24 May 2018.

## Background

Neuraxial procedures are commonly performed for therapeutic and diagnostic indications. These procedures are employed for surgical anesthesia, postoperative pain control, epidural labour analgesia and chronic pain management. More than 1.4 million Caesarean deliveries are performed in the United States with a majority using neuraxial anesthesia [1]. and more than 700,000 epidural procedures performed in 2006 [2, 3]. Epidural labour analgesia is utilized by increasing number of women in labour [4]. ,Therefore, it is essential to make neuraxial procedures safe and reliable.

Lumbar neuraxial procedures are typically performed via a ‘blind’ surface landmark and palpation guidance. Unfortunately, surface landmark identification may be highly inaccurate in identifying the underlying spinal structures [5, 6]. The identification of this space demands good knowledge of the anatomy and some skills due to its complexity. The failure in palpation from patient factors such as obesity, abnormal spine or previous spinal surgery results in difficult needle placement, leading to higher rate of complications. Permanent neurological injury may occur when spinal anesthesia is administered at a high spinal space [7]. Multiple attempts at neuraxial procedures could be associated with an increased risk of post-dural puncture headache, paraesthesia and spinal hematoma.

Neuraxial ultrasonography is a recent development in neuraxial anesthesia practice particularly in epidural space identification [8, 9]. It has been shown to be a safe and effective technique, with increasing use as an auxiliary tool to physical examination, enhancing the overall success rate of lumbar puncture and reducing the number of injection attempts. Even in normal surgical patients, the neuraxial anesthesia needle insertion first attempt success rate (success in achieving dural puncture on the first needle pass) is only about 50 to 60% when the palpation technique is used [10, 11].

However, despite its benefits and recommendations by international guidelines, ultrasound-guided neuraxial blocks are still not considered as routine clinical practice in many centres. A survey of 150 anesthesiologists in the United Kingdom showed that more than 90% of respondents have never used ultrasound for neuraxial blockade [12]. The reason is likely multifactorial, the most significant of which is that use of ultrasound for neuraxial blockade could be complex. Most of the clinical studies elucidating the benefits of ultrasound-guided neuraxial techniques originated from highly skilled operators, and learning and pattern recognition of spinal structures may be challenging especially in novice learners and even in those experienced operators when difficult spinal anatomy is present.

To fill this gap in current practice, we devised an intelligent image processing system with the ability to identify spinal landmarks in the ultrasound images [13–18]. In our preliminary studies done in a pilot proof of concept study in healthy volunteers, good accuracy in correct identification of L3/4 interspinous space in 93% of subjects (56 out of 60) was obtained. Primary inaccuracy was mainly due to the poor identification of the L5/S1 interspinous space. Hence, L2/3 instead of L3/4 interspinous space was identified. However, this had no implications on patient safety as spinal cord was above this level [16, 19]. We have further developed and refined the software to be used in a commercially available ultrasound machine (Sonosite M-Turbo Color Digital Ultrasound System).

We conducted a prospective cohort study with the primary aim of evaluating the first attempt success rate of spinal anesthesia using landmarks obtained from the novel automated spinal landmark identification technique. The primary hypothesis of the study was that automated spinal landmark identification algorithm using image processing system would achieve a mean 90% first attempt success rate of spinal anesthesia.

## Methods

The study was conducted at KK Women’s and Children’s Hospital and ethics approval was obtained (Singhealth Centralised Institutional Review Board: CIRB 2016/2262). The trial was registered on clinicaltrials.gov registry (NCT 03535155). Patients who met the inclusion criteria including women with age between 21 and 75 years old who required spinal anesthesia for surgical procedure, weight of 40-90 kg and height of 140-180 cm. The exclusion criteria included history of scoliosis, history of spinal instrumentation, drug allergy to ultrasound transmission gel and visible wound or injury in the lumbar spine. The patients were given the patient information sheet, before informed written consent was obtained from every patient by the investigators.

**Fig. 1:**
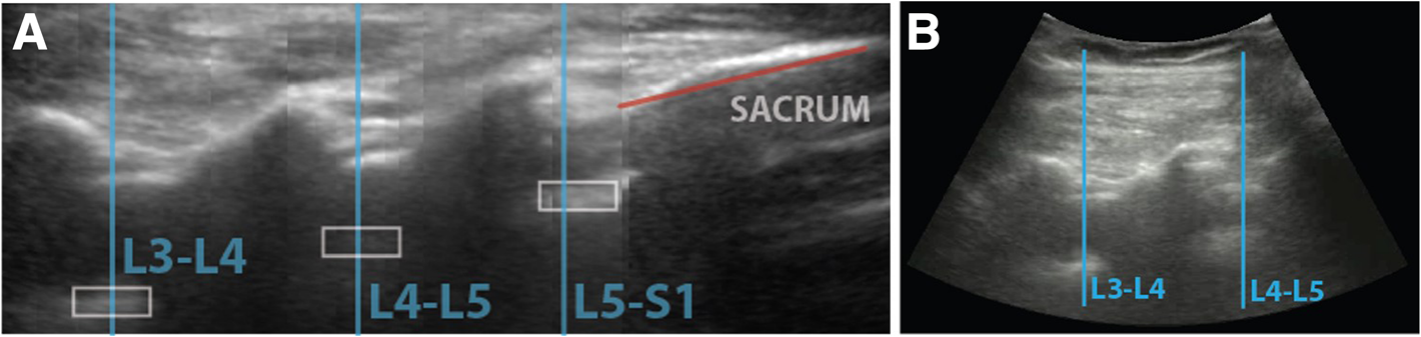
The step by step process of automated ultrasound spinal landmark identification.Please refer to the Methods, second paragraph

**Fig. 2:**
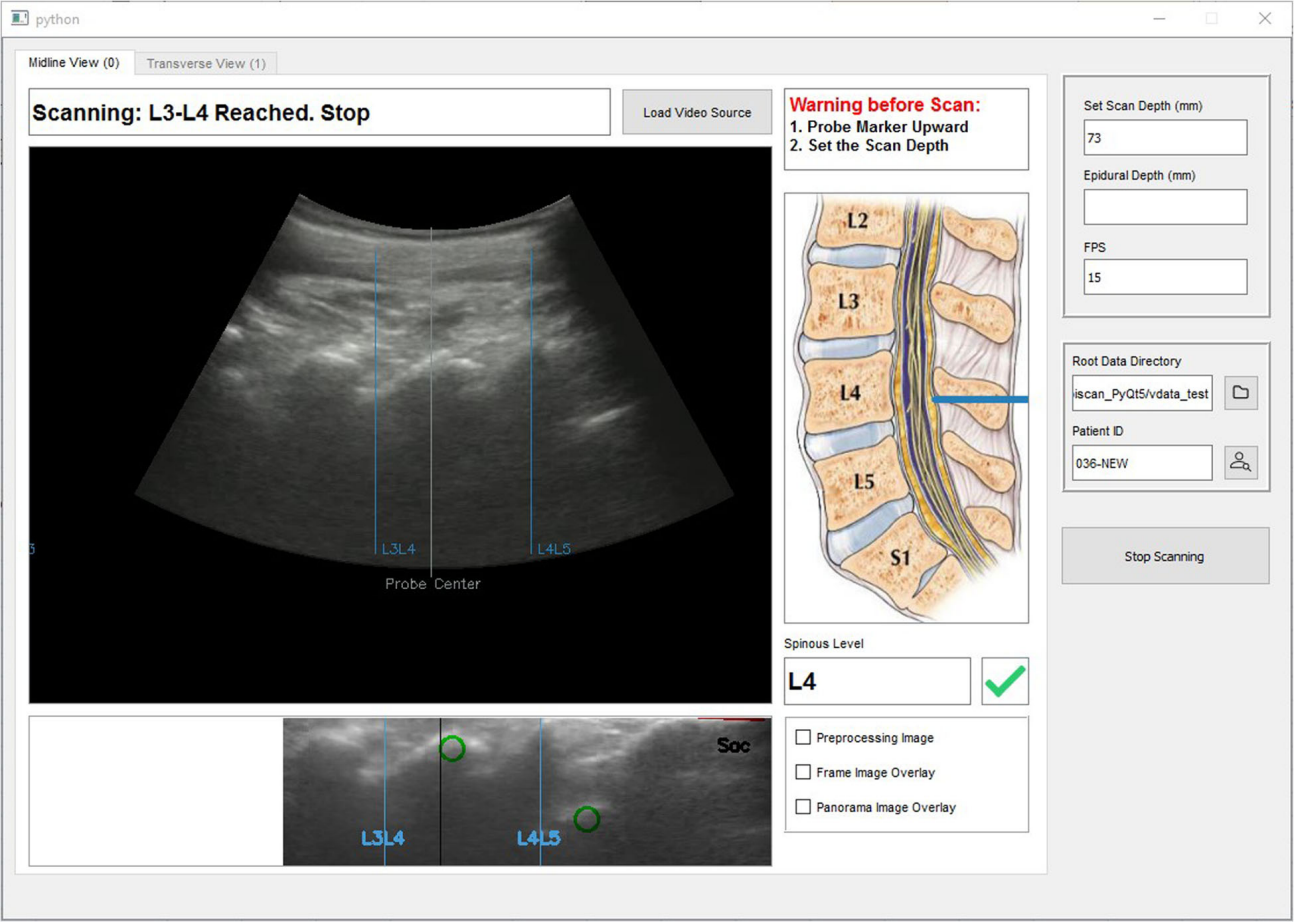
Midline View

**Fig. 3:**
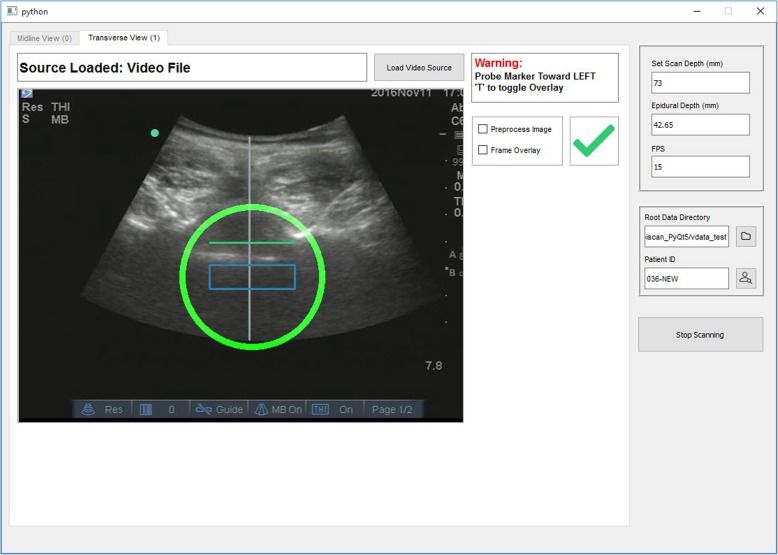
Transverse view

The patient assumed a seated position with the lower back exposed. Ultrasound gel was applied to the lower back before the investigator placed an ultrasound curved array probe around the sacral region. The graphical interface of the software, integrated with the ultrasound machine, guided the investigator to first identify the sacrum as a hyperdense line which was reflected as a computer marked red line as shown in Fig. 1a at the sacral region [20]. The investigator then moved the ultrasound probe in a steady vertical upward longitudinal direction of the lumbar spine and identified the lamina that were reflected as triangular peaks. Subsequently the laminas were identified and marked as rectangular white box (Fig. 1a). Upon identification of the L3/4 interspinous space, the system marked with a horizontal line along the midline of the probe by a surgical skin marker (Fig. 1b, Fig. 2). After the longitudinal section of the scan was completed, the investigator turned the probe 90 degrees clockwise around the probe centred to the transverse view. The transverse scan consisted of horizontal movements of the ultrasound probe along the previously marked line at the level of L3/4 by the investigator with minimal rotational movements to obtain the best view. The software program assists the operator in finding the best view- the appearance of a green tick on the screen indicates the achievement of a good view. The green tick would not appear if no good view can be obtained. The software would signal when the correct identification of the posterior complex was visualized. (Fig. 3) This position was then marked with a vertical line at the midline of the probe using a surgical skin marker. The program will only give instructions when all the anatomical landmarks are identified. After this scan sequence was completed, the anaesthetist used the identified needle entry insertion point to attempt spinal anesthesia insertion without traditional palpation. If the required dural puncture was not obtained at first attempt at the marked site, subsequent attempts could include the use of traditional palpation led skin surface markings. The number of spinal attempts was recorded and defined as the number of spinal needle insertion points on the skin.

Images produced were longitudinal and transverse images and videos of the scans, including the image of the L3/4 interspinous space in the longitudinal view and the posterior complex in the transverse view. The parameters in the image processing systems have undergone the offline training based on anatomical landmark images from patient’s database, hence less artefacts are less likely to affect the image processing system. All the identified images landmarks have been validated by the clinician investigators during the study.

Patient demographic data including age, weight, height and history of spine disorders were recorded. The number of spinal attempts and the time taken to identify the posterior complex in the transverse view were also recorded. The distance from skin to posterior complex was measured by the program. This was followed by the reading of the recorded scans by an experienced clinician investigator, blinded to the recorded images and videos by using study numbers, to determine the distance from skin to posterior complex from the scans. Congruency between the distance measured by the program and by the clinician investigator was then determined. The scans were done by only the principal investigator and co-investigator who are anesthesiology specialists. However, the needle insertions were done mostly by anesthesiology trainees who were assigned to the operating theatre as our center is a teaching hospital in obstetric anesthesia.

The planned sample size for the primary aim of the study was 100 subjects and it was calculated based on the following assumptions: expected first attempt spinal needle success rate of 90% using the automated spinal landmark identification system, a margin of error as 6.25% i.e. first attempt success rate between 83.5 to 96.0% and 95% confidence interval (95% CI) [21, 22]. Our pilot data showed that the accuracy of our system was 93% (56 out of 60 subjects) and we adjusted for 10% failure rate to obtain successful ultrasound imaging. We wanted to investigate this newer image processing system during this study. The primary outcome analysis was done using incidence proportion; with its corresponding 95% CI estimated using the Wilson score interval method for binomial data.

Primary outcome, *success at first attempt* at spinal needle insertion*,* was treated as binary data with status as “*yes*” or “*no*”. Success rate was expressed as proportion with corresponding 95% confidence interval (95%CI). Demographic and ultrasound imaging data were summarized based on status of success at first attempt. Continuous variables were summarized using mean standard deviation (SD) and median [interquartile range (IQR)] values while categorical variables were summarized as frequency (proportions). Pearson’s correlational and Cronbach’s coefficient alpha analysis were performed to assess internal reliability of program-recorded depth and the experienced clinician-measured depth to the posterior complex. SAS version 9.4 software (SAS Institute, Cary, North Carolina) was used for the analysis.

## Results

From May 2016 to May 2017, 100 patients who underwent spinal anesthesia for surgical procedure were recruited in the study. All the ultrasound imaging scans with automated spinal landmark identification were successfully performed. There were 99 patients who underwent Caesarean delivery and 1 patient underwent gynaecological procedure. Success rate for dural puncture at first attempt was 92% (95%CI 85–96%). Baseline characteristics in the group with success at first attempt and that with unsuccessful first attempt were similar (Table 1) In the group with unsuccessful first attempt (8/100 = 8%), 5 had dural punctures obtained at the second attempt, while 3 had dural punctures obtained at the third attempt. Median (IQR) time to detection of posterior complex was 45.0 [21.9, 77.3] secs.

**Table 1:** Demographic and clinical characteristics based on the success rates of the epidural insertion

	Success		Total *N* = 100
Variable	First Attempt *N* = 92	Not First Attempt *N* = 8	
Race, n (%)			
Chinese	51 (55.4)	05 (62.5)	056 (56.0)
Indian	12 (13.0)	00	012 (12.0)
Malay	12 (13.0)	01 (12.5)	013 (13.0)
Others	17 (18.5)	02 (25.0)	019 (19.0)
Age (Years), mean (SD)	33.5 (5.79)	36.4 (12.9)	033.7 (6.57)
Weight (kg), mean (SD)	69.2 (9.43)	66.0 (6.34)	69.0 (9.24)
Height (m), mean (SD)	1.6 (0.06)	1.6 (0.03)	1.6 (0.06)
BMI (kg/m^2^), mean (SD)	28.1 (3.16)	26.4 (2.34)	28.0 (3.13)
Level of scan operator Consultant, n (%)	92 (100)	8 (100)	100 (100)
Skin to posterior complex depth (mm), mean (SD)	44.7 (6.3)	39.6 (6.7)	44.3 (6.5)

**Fig. 4:**
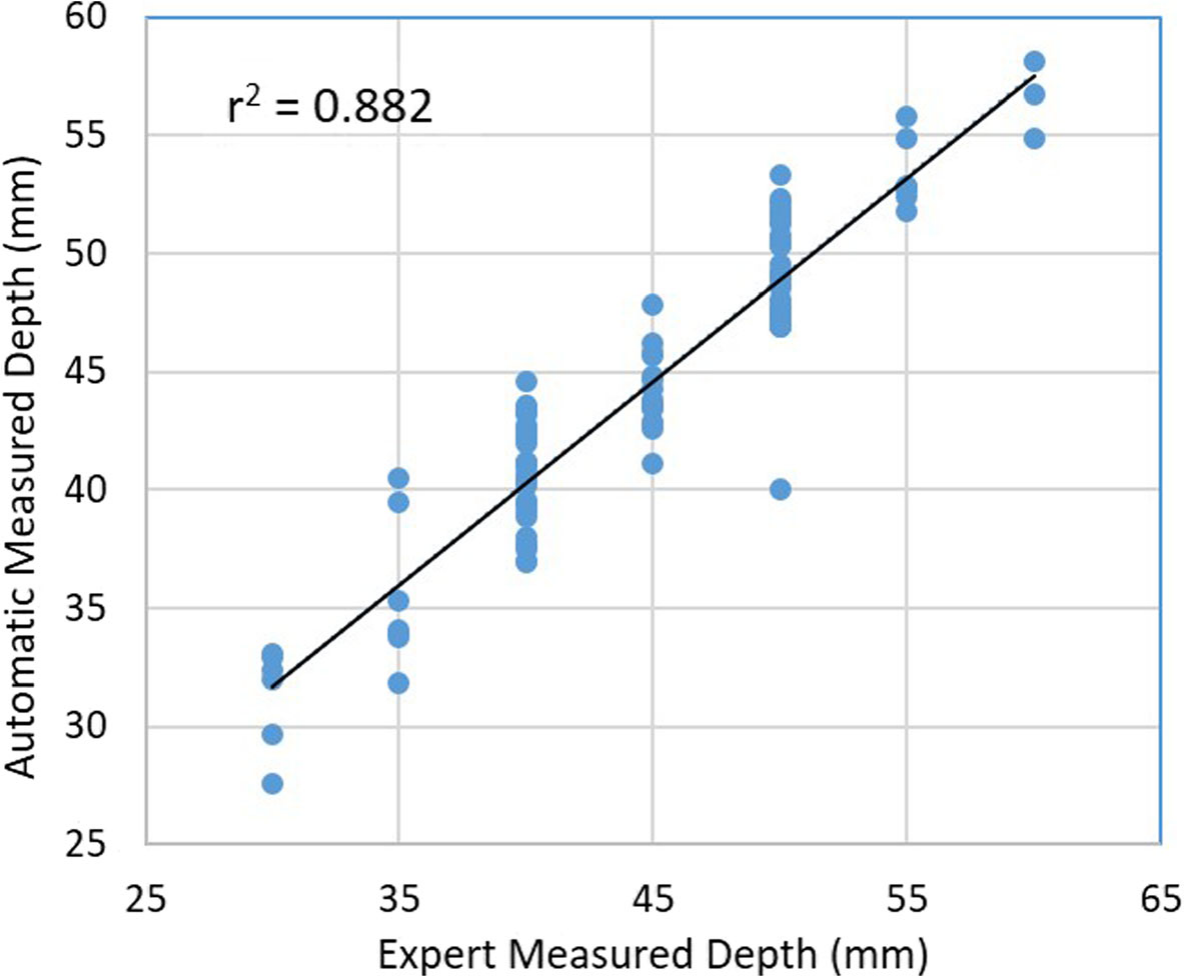
Pearson’s correlation between program-recorded depth and the experienced clinician- measured depth to the posterior complex

The mean (SD) number of attempts needed to scan the lumbar area until obtaining the L3/4 level was 3.1 (3.0). There is good correlation observed between the program-recorded depth and the experienced clinician- measured depth to the posterior complex. The Pearson’s correlation and Cronbach’s alpha was 0.94 and 0.97 respectively (Fig. 4).

## Discussion

Ninety two (92 of 100) patients had successful first attempts and all ultrasound imaging scans with automated spinal landmark identification were successful. The median (IQR) time to detection of posterior complex was 45.0 [21.9, 77.3] secs. Good correlation was observed between the program-recorded depth and the experienced clinician investigator-measured depth to the posterior complex.

The successful first attempt rate in neuraxial anesthesia is higher than 61.6% described by de Filho et al. when palpation directed surface landmarking was employed in a population that was similar in demographics of age and BMI [10]. In addition, 99% of our patients (99 of 100) recruited were obstetric cases that could pose a more challenging anatomy for neuraxial techniques. The high first attempt success rate could reduce complications associated with multiple attempts such as patient discomfort, increased incidence of post-dural puncture headache, paraesthesia and spinal hematoma. Patient safety could be improved as this automated ultrasound spinal landmark identification system allows for correct identification of spinal structures in particular the spinal level of needle insertion.

Ultrasound imaging may be especially useful for difficult patients with obesity, abnormal spinal anatomy and previous spinal surgery where palpation of spinal landmarks can be challenging [23]. In patients with abnormal spinal anatomy, ultrasound imaging has been shown to improve the neuraxial anesthesia needle insertion first attempt success rate from 32% using the palpation technique to 65% with the use of ultrasound imaging by Chin KJ et al [23]. Furthermore, lumbar ultrasonography has been recommended for clinical use when performing neuraxial anesthesia by the National Institute for Health and Care excellence (NICE) guidelines and systematic review [24, 25].

We are evaluating if identification of the site of needle insertion will improve successful needle insertion with the first attempt. Often, especially with junior trainees or in patients with more challenging anatomy, the wrong identification of site of needle insertion is a significant contribution to the inability to obtain a successful needle insertion with the first attempt. The utility of this automated spinal landmark identification is to circumvent errors in identifying site of needle insertion and henceforth, improve successful needle insertion with the first attempt.

Poor uptake to ultrasound guided neuraxial techniques could in part be due to the lack of technical skills in identifying the anatomical landmarks and the perceived belief that using an ultrasound to guide neuraxial techniques may be too time-consuming compared to the traditional palpation led surface landmarking technique. The automation in this novel system could allow operators to have confirmation of the sonographic images and structures. This allows for both novice trainees and experienced clinicians who are unfamiliar with ultrasound techniques to be able to harness the benefits of ultrasound-guided neuraxial techniques. This study showed that using ultrasound would not compromise on procedural time as the time taken to obtain the surface landmark with the automated ultrasound-guided neuraxial technique was under a minute. Furthermore, the high rate of first attempt success rate could potentially reduce the complications caused by multiply entry attempts.

### Limitations of this study

Limitations of this study would include a lack of a comparator arm. However, we were investigating a novel automated spinal landmark system and future studies with a randomized trial design would be planned. In addition, the limitation of the proposed image processing program is the high sensitivity required of quality of ultrasound images. However, it is crucial to achieve a high accuracy (less false positives) at the sacrifice of non-optimal recall rate. This may lead to possible additional attempts in scanning as the algorithm is highly specific to only accept given information when all landmarks are detected. The system is validated by our study population (young obstetric women with BMI below 30 kg/m^2^) and it is not designed or validated by complex spinal anatomy, obesity patients, paediatric patients and geriatric patients. As the software program requires first identifying the sacrum and then counting the spinal level till L3/4. The abnormal anatomy such as fusion or reduced interspinous distance could increase the risk of misinterpretation.

We chose our primary aim to evaluate the clinical relevance of the automated ultrasound guided system as we had previously evaluated the correlation between spinal landmark identified by the automated machine and identified by an expert anesthesiologist skilled in spine imaging in our preliminary study, which had showed a 93% correlation [13]. We agree that the image processing system does not improve operator error in needle insertion technique, but we are evaluating how the automated ultrasound guided technique can improve first pass attempts despite variations in operator errors in needle insertion technique.

### Future directions

We observed a good correlation was observed between the program-recorded depth and the experienced clinician-measured depth to the posterior complex. This would be useful in future applications of using the program to guide epidural insertion. Its clinical correlation and applicability can be investigated in subsequent studies where congruency between distance to epidural space measured by the program against that measured by the epidural needle during epidural insertion. Hence, future work would be to investigate the correlation between program-recorded depth to the posterior complex and the actual distance to epidural space during epidural catheter insertion.

We plan to further determine the accuracy of locating the spinal level and the success of needle insertion by anesthesia trainees, and investigate the use of this system in the obese population, where this automated ultrasound guided neuraxial technique would be more useful, as surface landmarks for neuraxial anesthesia could be more challenging.

## Conclusions

This study found that the use of this novel automated ultrasound-guided surface landmark system is a promising option to assist clinicians in improving identification of spinal landmarks, which could contribute to the high first attempt success of spinal anesthesia with acceptable procedural scan time.

## Abbreviations

BMI: Body mass index
CI: Confidence interval
CIRB: Centralised Institutional Review Board
IQR: Interquartile range
NICE: National Institute for Health and Care excellence
SD: Standard deviation
